# A Screen of Traditional Chinese Medicinal Plant Extracts Reveals 17 Species with Antimicrobial Properties

**DOI:** 10.3390/antibiotics13121220

**Published:** 2024-12-17

**Authors:** Garrett L. Ellward, Macie E. Binda, Dominika I. Dzurny, Michael J. Bucher, Wren R. Dees, Daniel M. Czyż

**Affiliations:** Department of Microbiology and Cell Science, College of Agricultural and Life Sciences, University of Florida, Gainesville, FL 32611, USA; glee.ellward@ufl.edu (G.L.E.); dominika.dzurny@ucf.edu (D.I.D.);

**Keywords:** medicinal plants, traditional Chinese medicine, phytochemicals, antimicrobials, antibiotic resistance

## Abstract

**Background/Objectives:** Antimicrobial resistance (AMR) is a growing threat that undermines the effectiveness of global healthcare. The Centers for Disease Control and Prevention and the World Health Organization have identified numerous microbial organisms, particularly members of the ESKAPEE pathogens, as critical threats to global health and economic security. Many clinical isolates of these pathogens have become completely resistant to current antibiotics, making treatment nearly impossible. Herbal remedies, such as those found in Traditional Chinese Medicine (TCM), have been practiced for thousands of years and successfully used to treat a wide range of ailments, including infectious diseases. Surprisingly, despite this extensive knowledge of folk medicine, no plant-derived antibacterial drugs are currently approved for clinical use. As such, the objective of this study is to evaluate the antimicrobial properties of extracts derived from TCM plants. **Methods:** This study explores a comprehensive library comprising 664 extracts from 132 distinct TCM plant species for antimicrobial properties against gram-negative (*Escherichia coli*) and gram-positive (*Micrococcus luteus*) bacteria using liquid and solid in vitro assays. **Results:** Intriguingly, our results reveal 17 plant species with potent antimicrobial properties effective primarily against gram-positive organisms, including *Streptococcus aureus* and *epidermidis*. A literature search revealed that nearly 100 purified compounds from the identified TCM plants were previously isolated and confirmed for their antimicrobial properties, collectively inhibiting 45 different bacterial species. **Conclusions:** Our results indicate that phytobiotics from the identified plants could serve as potential candidates for novel antimicrobials.

## 1. Introduction

Globally, antimicrobial resistance (AMR) has emerged as a significant threat to sustainable healthcare. In the United States alone, the Centers for Disease Control and Prevention attributes over 35,000 deaths annually to AMR, with nearly 5 million individuals worldwide succumbing to antimicrobial-resistant infections [1,2]. These infections not only cause a significant loss of life, but also result in economic losses amounting to billions of dollars [3]. Without viable alternatives to current therapies, it is estimated that the worldwide death toll could increase nearly tenfold, while the economic impact could reduce the global gross domestic product by more than 3% [3,4]. Some organisms in particular, such as the ESKAPEE pathogens, named for *Enterococcus faecium*, *Staphylococcus aureus*, *Klebsiella pneumoniae*, *Acinetobacter baumannii*, *Pseudomonas aeruginosa*, *Enterobacter* spp., and *Escherichia coli*, are labeled as critical or high-priority threats to global health security due to their prevalence in nosocomial infections and high rates of antimicrobial resistance [1,5,6,7]. As the number of AMR bacterial strains continues to rise, the development of new antibiotics has nearly halted, primarily due to the unfavorable economics associated with sales of therapeutics for acute conditions such as infections [8].

Without new antibiotic development, the research has turned its attention to investigating alternative therapies, including drug repurposing, bacteriophages, silver nanoparticles, and other options [9,10,11,12,13]. Herbal remedies within Traditional Chinese Medicine (TCM) offer a promising yet underexplored source of potential antimicrobial drugs. A comprehensive collection of 664 extracts from 132 TCM plant species is available from the National Cancer Institute [14]. Research into TCM plants and their potential antimicrobial properties has significantly increased over the past several years. While many of the TCM plants have been used for medicinal purposes for thousands of years, demonstrating efficacy against various ailments, including infectious diseases [15], the active components are emerging as potential therapeutics [16]. Plant-derived compounds have long been known for their antimicrobial properties, primarily attributed to organic acids, polyphenols, alkaloids, flavonoids, quinones, and volatile oils [17,18,19,20,21]. The most notable of these applications is perhaps artemisinin, derived from *Artemisia annua*, and is used against *Plasmodium falciparum* and related species [22]. Discovered by Professor Youyou Tu, artemisinin has become the standard treatment for many forms of malaria and has revolutionized healthcare, earning Professor Tu the Nobel Prize in 2015 [23].

While plants produce thousands of compounds that enhance their immune systems and protect against bacterial infections, efforts to identify phytobiotics suitable for human use have been challenging, as plant compounds are often significantly weaker than antimicrobials produced by bacteria and fungi [24]. Furthermore, it is believed that, in some cases, plant extracts are only effective in conjunction with other compounds derived from the same plant, as such combinations are better suited to overcoming bacterial resistance [24]. Among other challenges, plant-derived metabolites often have complicated biosynthesis pathways, which hinders their scale production [25,26]. Despite all the complexities, plants remain a promising source of novel antimicrobials.

In this study, we examine extracts from the TCM library for their antibacterial activity against gram-positive (*Micrococcus luteus*, *S. aureus*, and *Staphylococcus epidermidis*) and gram-negative (*E. coli*) organisms. Employing a combination of solid and liquid screening assays, we initially tested each of the 664 extracts against *M. luteus* and *E. coli* and followed up by confirming their antimicrobial activity using *Streptococcus* species. Of the 664 extracts tested, 25 from 17 unique plant species demonstrated activity against gram-positive bacteria. Our study provides a comprehensive analysis of TCM plants for antimicrobial activity, reveals potentially new candidates for further therapeutic development, and creates new opportunities for applications in human and animal medicine.

## 2. Results

### 2.1. Experimental Overview and Identification of TCM Plant Extracts with Antimicrobial Properties

**Figure 1:**
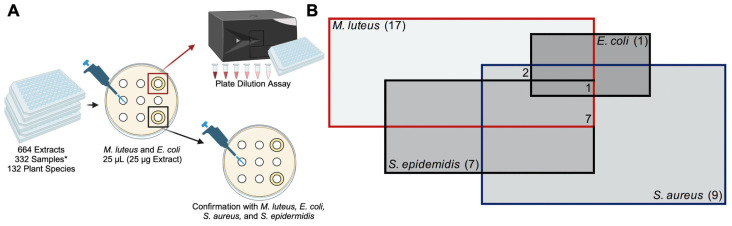
Overview of the screening process for antimicrobial phytobiotics from the TCM library. (**A**) The TCM library consisted of 664 extracts * (332 aqueous and 332 organic) derived from 132 unique plant species. First, 25 µL of each extract was applied onto plain paper discs placed on freshly spread lawns of *M. luteus* (SK58) or *E. coli* (DH5a). Following incubation, the plates were inspected for zones of inhibition. Extracts that showed inhibition were subjected to serial dilution and further testing using a liquid plate dilution assay to assess their antimicrobial potency. Antimicrobial activity was confirmed against *E. coli*, *M. luteus*, *S. aureus*, and *S. epidermidis* for all extracts that initially produced a zone of inhibition. (**B**) Venn diagram representing the distribution of 17 unique plant species and their inhibitory effect on each of the bacterial strains tested.

To identify plants with antimicrobial properties, we employed both solid and liquid assay screens targeting representative gram-positive and gram-negative organisms. Initially, 25 µL (25 µg total) of each of the 664 extracts was spotted onto a sterile paper disc and incubated on a bacterial lawn of two indicator strains, *E. coli* (DH5α) or *M. luteus* (SK58). We specifically chose these strains of bacteria based on our preliminary assessment, indicating that they are more susceptible to antibiotics compared to other strains, thereby enhancing the sensitivity of our screen. Collectively, we tested 768 spots that included vehicle controls and 664 extracts. As expected for a gram-negative strain, *M. luteus* was much more susceptible to the antimicrobial activity of extracts than *E. coli*. Therefore, we repeated the screen of all extracts using *M. luteus*. Collectively, of all the extracts tested, 25 exhibited inhibitory properties against gram-positive bacteria, and one showed mild inhibition against gram-negative *E. coli* (Figure 1 and Figure S1, Table 1). Extracts that produced a zone of inhibition (ZOI) greater than the diameter of the paper disc (6 mm) were further validated using a disc diffusion assay and subsequently tested in a plate dilution assay against a broader selection of bacterial species.

### 2.2. Extract Screening Reveals 17 TCM Plant Species with Antimicrobial Properties

**Figure 2:**
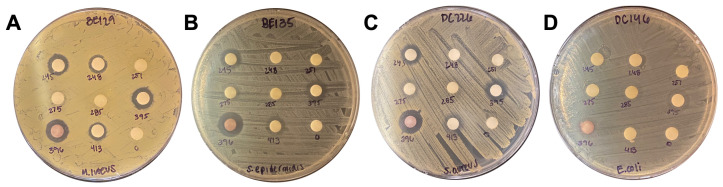
Disc diffusion assay of TCM library. Representative images of the TCM library extracts spotted against (**A**) *M. luteus*, (**B**) *S. epidermidis*, (**C**) *S. aureus*, and (**D**) *E. coli*. Zones of inhibition were measured for every effective extract.

To further validate the efficacy of the 25 extracts with antimicrobial activity, we repeated the disc diffusion assay using *M. luteus* SK58, *S. epidermidis* BCM0060, *S. aureus* 12600, and *E. coli* DH5*a*. All ZOI measurements from both the initial and confirmational disc diffusion assays were recorded and are shown in Table 1, with full information, including extract IDs, listed in Table S1. For *M. luteus*, we recorded the largest ZOI measurement across the three runs. These experiments reveal several extracts with robust antimicrobial properties. In particular, the *Lithospermum erythrorhizon* extract (#396) was the only one that inhibited all four species, though extract #460 from the same plant inhibited three bacterial strains (Figure 2, Table 1). While the #396 extract robustly inhibited gram-positive bacteria, it exhibited minimal inhibition against *E. coli * (Figure 2 and Figure S2). *Evodia rutaecarpa* extracts (#33, #424, and #727), *Glycyrrhiza uralensis* extract (#245), *Juncus effusus* extracts (#275, #395, and #415), *Sophora flavescens* extract (#413), *Agrimonia pilosa* extract (#456), and *Salvia miltiorrhiza* extract (#694) inhibited all gram-positive bacteria tested (Table 1). The extracts from all 17 plants identified in our study were previously extensively studied for their antimicrobial properties against a broad spectrum of bacterial pathogens (Table 2), further supporting their antimicrobial properties.

**Table 2:** Summary of the 17 TCM plant species with documented antimicrobial properties. Overlapping antimicrobial activity observed for specific bacterial strains in our screen is highlighted in bold. Selected publications that describe targeted bacteria are listed. * The families *Compositae* and *Asaraceae* refer to the same family.

* Family.	Genus	Species	Common Name	Chinese Name	Tested for Antimicrobial Activity [Citation]
* Compositae *	* Asarum *	* heterotropoides *	Wild Ginger	細辛	*Ralstonia solanacearum*, *Xanthomonas oryzae*, *Pseudomonas syringae*, *Xanthomonas axonopodis* [27]; *Fusobacterium nucleatum*, *Prevotella intermedia*, *Porphyromonas gingivalis* [28]. *Clostridioides difficile*, *Clostridium paraputrificum*, *Clostridium perfringens*, ***Staphylococcus aureus***, *Bacteroides fragilis*, *Escherichia coli*, *Salmonella enterica* serovar Typhimurium [29]; *Listeria monocytogenes* [30]; ***Staphylococcus epidermidis***, ***Micrococcus luteus***, *Corynebacterium jeikeium*, *Corynebacterium xerosis*, *Propionibacterium freudenreichii* [31]
* Boraginaceae *	* Lithospermum *	* erythrorhizon *	Purple Gromwell	紫草	*Bacillus subtilis*, *Bacillus thuringiensis*, *Clavibacter michiganensis*, *Agrobacterium radiobacter*, *Agrobacterium rhizogenes*, *Agrobacterium tumefaciens*, *Bulkholderia cepacian*, *Erwinia herbicola*, *Ralstonia solanacearum* [32]; ***Staphylococcus aureus*,** *Micrococcus roseus***, *Micrococcus luteus*, ***Bacillus subtilis* [33]
* Compositae *	* Tussilago *	* farfara *	Coltsfoot	款冬花	*Bacillus cereus*, *Staphylococcus aureus* [34]; *Pseudomonas aeruginosa*, *Escherichia coli*, *Staphylococcus aureus* [35];. *Escherichia coli*, *Klebsiella pneumoniae*, *Salmonella enterica*, *Shigella sonnei*, *Yersinia enterocolitia*, *Bacillus thuringiensis*, *Clostridium perfringens*, *Haemophilus influenzae*, *Listeria monocytogenes*, *Staphylococcus aureus* [36]; *Escherichia coli*, *Serratia rubidaea*, *Staphylococcus epidermis*, *Lactobacillus rhamnosus*, *Pseudomonas aeruginosa*, *Enterococcus raffinosus* [37]
* Compositae *	* Echinops *	* latifolius *	Globe Thistle	驴欺口	None
* Compositae *	* Artemisia *	* annua *	Sweet Wormwood	青蒿	*Haemophilus inflenzae*, *Enterococcus faecalis*, *Streptococcus pneumoniae*, ***Micrococcus luteus*** [38]; *Bacillus cereus*, *Staphylococcus aureus*, ***Micrococcus luteus***, *Escherichia coli*, *Klebsiella pneumoniae*, *Salmonella enteritidis*, *Shigella* sp. [39]; *Staphylococcus aureus*, *Escherichia coli*, *Bacillus subtilis*, *Bacillus thuringiensis* [40]; *Enterococcus hirae* [41]; *Staphylococcus aureus*, *Escherichia coli* [42]; *Staphylococcus aureus*, *Escherichia coli*, *Bacillus cereus*, *Enterococcus faecalis*, *Pseudomonas aeruginosa* [43]; *Staphylococcus aureus*, *Bacillus subtilis*, *Bacillus pumilus*, *Bacillus cereus*, *Micrococcus luteus*, *Escherichia coli*, *Salmonella typhi*, *Pseudomonas aeruginosa* [44]
* Compositae *	* Artemisia *	* argyi *	Mugwort	艾草	*Staphylococcus aureus*, *Escherichia coli*, *Bacillus subtilis*, *Listeria monocytogenes*, *Pseudomonas aeruginosa*, *Streptococcus pneumoniae*, *Proteus mirabilis*, *Enterococcus faecalis*, *Streptococcus agalactiae* [45]; *Staphylococcus aureus*, *Bacillus subtilis*, *Listeria monocytogenes*, *Escherichia coli*, *Proteus vulgaris*, *Salmonella enteritidis* [46]
* Compositae *	* Sigesbeckia *	* orientalis *	St. Paul’s Wort	豨莶	*Staphylococcus aureus*, *Pseudomonas aeruginosa*, *Escherichia coli* [47]; *Bacillus subtilis*, *Staphylococcus epidermidis*, *Staphylococcus aureus*, *Streptococcus oralis*, *Acinetobacter baumannii*, *Escherichia coli*, *Pseudomonas aeruginosa* [48]
* Dioscoreaceae *	* Dioscorea *	* nipponica *	Japanese Yam	穿龙薯蓣	*Bacillus subtilis*, *Staphylococcus aureus*, *Proteus vulgaris*, *Salmonella* Typhimurium [49]
* Juncaceae *	* Juncus *	* effusus *	Soft Rush	灯心草	***Staphylococcus aureus***, *Bacillus subtilis* [50]; ***Micrococcus luteus***, *Bacillus subtilis*, ***Staphylococcus aureus*** [51]
* Lamiaceae *	* Pogostemon *	* cablin *	Patchouli	广藿香	*Staphylococcus aureus*, *Escherichia coli*, *Pseudomonas aeruginosa*, *Bacillus subtilis*, *Staphylococcus epidermidis*, *Streptococcus oralis*, *Streptococcus pneumoniae*, *Streptococcus constellatus*, *Streptococcus pyogenes*, *Streptococcus mitis* [52]; *Staphylococcus aureus*, *Shigella* sp. [53]
* Labiatae *	* Salvia *	* miltiorrhiza *	Red Sage	丹参	*Agrobacterium tumefaciens*, *Escherichia coli*, *Pseudomonas syringae*, *Ralstonia solanacearum*, *Xanthomonas vesicatoria*, *Bacillus subtilis*, ***Staphylococcus aureus***, *Staphylococcus haemolyticus* [54]
* Fabaceae *	* Glycyrrhiza *	* uralensis *	Chinese Licorice	甘草	*Streptococcus mutans* [55]; ***Staphylococcus aureus*** [56]
* Fabaceae *	* Sophora *	* flavescens *	Sophora Root	苦参	***Staphylococcus aureus***, *Bacillus subtilis*, *Salmonella* Typhimurium, *Proteus vulgaris*, *Escherichia coli* [57]; *Streptococcus mutans* [58]
* Compositae *	* Areca *	* catechu *	Areca Palm	檳榔	*Bacillus subtilis*, *Staphylococcus aureus* [59]
* Rosaceae *	* Agrimonia *	* pilosa *	Chinese Agrimony	龙芽草	*Listeria monocytogenes*, *Streptococcus enteritidis*, *Escherichia coli* [60]
* Rubiaceae *	* Rubia *	* cordifolia *	Indian Madder	茜草	*Erwinia herbicola*, *Agrobacterium tumefaciens*, *Xanthamonas campestris* [61]; *Bacillus cereus*, *Bacillus pumilus*, *Bacillus subtilis***, *Micrococcus luteus***, *Mycobacterium luteum*, *Staphylococcus aureus*, *P. aeruginosa* [62]
* Rutaceae *	* Evodia *	* rutaecarpa *	Evodia Fruit	吳茱萸	*Escherichia coli*, ***Staphylococcus aureus*** [63]; ***Staphylococcus aureus***, ***Staphylococcus epidermidis***, *Bacillus subtilis* [64]

### 2.3. Liquid Assay

**Figure 3:**
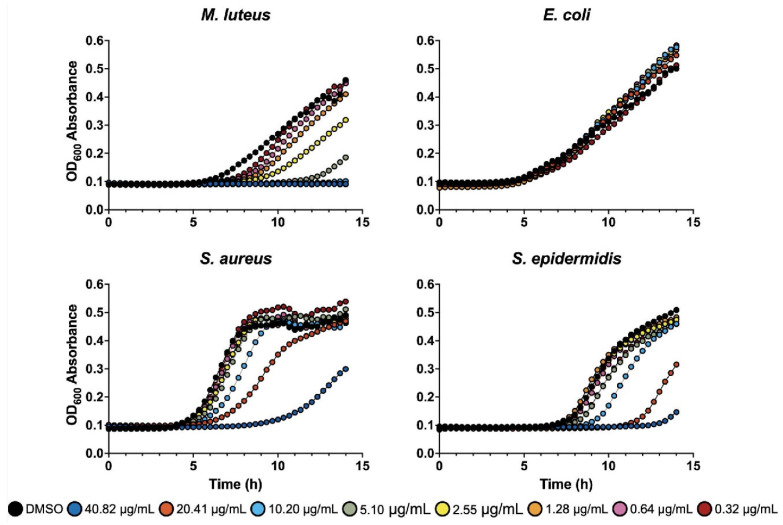
Antimicrobial assessment of *E. rutaecarpa* extract (#727) using the plate dilution liquid assay. The extract exhibited an inhibitory effect against all gram-positive bacteria tested. The MIC for *M. luteus* was 10.2 µg/mL. No other strain was fully inhibited, though #727 did significantly affect the microbial growth of *S. aureus* and *S. epidermidis*. Control represents vehicle DMSO.

*E. rutaecarpa* extract (#727) was exceptionally effective against *M. luteus* and other gram-positive species (Table 1, Figures S1 and S2). Additionally, three different extracts from this plant (#33, #424, and #727) were identified as hits in the initial screen (Table S1), further confirming its antimicrobial potential. Based on these findings, we selected the *E. rutaecarpa* extract #727 for further evaluation of its efficacy in a plate dilution liquid assay. The extract was serially diluted and tested for its ability to inhibit the growth of the four bacterial strains. Our results reveal that the extract was the most effective against *M. luteus*, with an MIC of 10.2 µg/mL (Figure 3). Although the extract did not fully inhibit other gram-positive strains, it still exhibited significant antimicrobial activity with a greater than 50% growth inhibition observed (Figure 3). In total, we identified 25 extracts from 17 unique plant species that exhibited antimicrobial properties, primarily against gram-positive bacteria. Since crude extracts, rather than isolated active components, were tested in our assays, the exact potency of each phytobiotic remains to be determined in follow-up studies.

### 2.4. Phylogenetic Analysis

**Figure 4:**
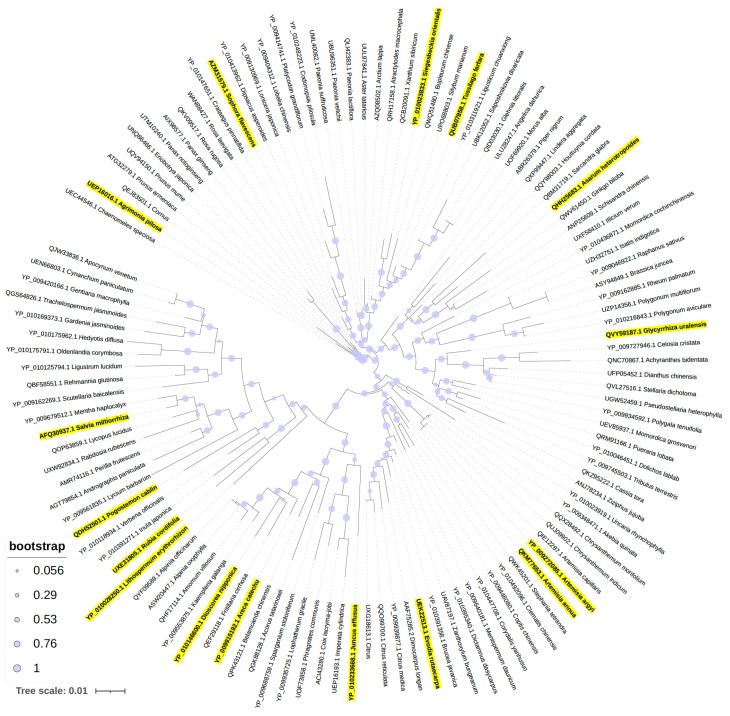
Phylogenetic analysis of TCM library constructed using RuBisCO. Hits from the library represent a diverse selection of genera. Highlighted species represent plants with antimicrobial properties identified in our study. The scale bar represents the evolutionary distance between species based on RUBisCO sequences.

We sought to examine potential evolutionary relationships between the plants that exhibited antimicrobial properties of their extracts. To accomplish this, we constructed a phylogenetic tree based on the ribulose bisphosphate carboxylase/oxygenase (RuBisCO) protein sequences from 118 plant species with available sequence data (Figure 4). Of the 17 plants identified for antimicrobial properties, 16 had RuBisCO sequences available from the National Center for Biotechnology Information (NCBI) database, and these plant species were highlighted in yellow on the phylogenetic tree. Notably, *Artemisia*, a genus known to produce the antimalarial compound artemisinin, was the only genus with multiple effective extracts from different species clustering together on the phylogenetic tree. Interestingly, *L. erythrorhizon* and *R. cordifolia* were also closely positioned, however they have distinct evolutionary lineages as they belong to different orders and families. Overall, this analysis suggests no clear evolutionary link among the 17 plants with available protein sequences; however, further exploration involving whole-genome analyses could potentially uncover more genetic similarities between these plants.

### 2.5. Active Components of TCM Plant Extracts

Numerous studies have isolated active components from almost all the TCM plants identified in our study, yielding at least 99 compounds with antimicrobial activity against 45 unique bacterial species, including all ESKAPEE pathogens (Table S2) [27,29,33,39,47,48,50,51,63,65,66,67,68,69,70,71,72,73,74,75,76,77,78,79,80,81,82,83,84]. Using 78 unique and available compound identification (CID) numbers obtained from PubChem, we determined that 64 of the previously isolated small molecules satisfy the Lipinski Rule of Five, which predicts oral bioavailability (Table S3). As such, the potential for these antimicrobial medicinal plants to retain their activity when consumed whole, or as herbal or other extracts, is likely maintained.

To further analyze the antimicrobial components, we analyzed the compounds for common core structures. To accomplish this, we retrieved Simplified Molecular Input Line Entry System (SMILES) strings for each available CID (Table S3), clustered the compounds based on Tanimoto similarity scores using ChemMine tools, and visualized the results using iTol (Figure S3). To identify common structures, we chose a 0.7 cutoff to allow for a moderate degree of structural similarity, often used to broadly group compounds and allow for more diversity within each cluster. We identified ten significant clusters at the chosen cutoff. Almost all compounds that clustered together were extracted from the same plant. For example, clusters 1–6 and 9–10 contain several structurally similar compounds. However, there were a few exceptions, such as cluster 7, which contained a-terpineol and a-terpinol, two compounds that were extracted from *A. annua* and *A. argyi*, respectively (Figure S3, Table S2). These two plant species also share structurally similar borneol and L-borneol found in cluster 8 and both synthesize caryophyllene oxide and camphor. Furthermore, 1,8-cineole was synthesized by *A. heterotropoides* and *A. annua*, two plants from the same family but different genera (Table S2). As expected from the high Tanimoto similarity score cutoff, the structural analysis of the compounds across the ten clusters revealed some common scaffolds (Figure S4).

Collectively, while nearly 100 known antimicrobial compounds were isolated from each of the 14 plants to our knowledge, no known studies fractionated compounds from *E. latifolius*, *D. nipponica*, or *R. cordifolia*. More studies, especially in vivo, are needed to assess the antimicrobial efficacy of the active components.

## 3. Discussion

### 3.1. TCM Plant-Derived Antimicrobials

An estimated 50% of pharmaceuticals on the market is derived from plants; however, none of these drugs are antimicrobials [85]. Such a lack of plant-derived antimicrobials is primarily attributed to numerous challenges [24,25,26]; nonetheless, plants remain a promising source of novel antimicrobials [86]. In a screen of 664 extracts from 132 unique TCM plants, we identified 25 extracts from 17 unique species that exhibit antimicrobial properties, which is close to a 13% success rate. This number is high compared to previous estimates of the prevalence of plant species with antimicrobial properties, which reveal a rate of less than 1% [87]. The extracts included within the NCI collection represent the most commonly used TCM plants [14,88]; therefore, the active components have been selected for centuries for medicinal indications across various ailments, including infections. The antimicrobial properties of the 17 plant species are further supported by extensive studies revealing their potential utility against a broader spectrum of bacterial strains (Table 2). Of the 17 plants, all but one, *Echinops latifolius*, have been previously noted for their antimicrobial activities (Table 2).

### 3.2. Antimicrobial Activities of TCM Plant Extracts

While all of the effective extracts had an inhibitory effect against *M. luteus* and other gram-positive strains, only one, *L. erythrorhizon*, resulted in a mild inhibition of *E. coli*. *L. erythrorhizon* has been previously investigated for its antimicrobial properties, with several studies demonstrating its potency against gram-positive and gram-negative bacteria (Table 2). *L. erythrorhizon* is known to produce shikonin, a purple dye that exhibits antimicrobial activity against *E. coli* [89], *S. aureus* [90], and other microbes, including fungi [32,33]. Moreover, shikonin is known to synergize with antibiotics, such as colistin [91], and other clinically important antimicrobials [92], but its activity remains predominantly effective against gram-positive bacteria [32,33]. The lack of efficacy against gram-negative bacteria among the identified plants does not negate the potential of their extracts to reveal novel antimicrobials, as the purified active component will likely exhibit increased potency alone or, as demonstrated by previous research, in combination with traditional antibiotics. Numerous studies isolated active fractions from the 17 identified plants effective against gram-negative bacteria (Table 2).

Several extracts present in the library that originated from the same plant species were identified in our screen. These included *E. rutaecarpa*, *Rubia cordifolia*, *Dioscorea nipponica*, *A. argyi, J. effusus*, and *L. erythrorhizon* (Table 1). Notably, *E. rutaecarpa* was effective against all gram-positive bacteria tested and completely inhibited *M. luteus* in a plate dilution assay at a concentration of 10.2 µg/mL. Evodiamine is the active component of *E. rutaecarpa*, typically sourced from its fruit and recognized for its potent anti-inflammatory [93], antimicrobial [94], and anticancer properties [95]. However, evodiamine is not the plant’s only active component. Liang et al. highlighted limonin, another major component of *E. rutaecarpa* extract, as having a stronger antimicrobial effect compared to evodiamine, showing bacteriostatic activity towards *E. coli* and *S. aureus* at concentrations of 0.11 mg/mL and above [63]. Our results reveal that the crude *E. rutaecarpa* extract can inhibit bacteria at much lower concentrations, albeit not against gram-negative bacteria. The MIC of evodiamine against *Helicobacter pylori* was 20 µM (6.07 µg/mL) [94], which also seems high compared to the MIC of 10.2 µg/mL we obtained using the crude extract. Wang et al. reported that *E. rutaecarpa* quinolone alkaloids exhibited antimicrobial activity approaching that of ciprofloxacin, with 4–8 µg/mL MICs against *S. aureus*, *S. epidermidis*, and *B. subtilis* [64]. Given that *E. rutaecarpa* synthesizes at least 11 potent antimicrobials (Table S2), it is unlikely that the antimicrobial activity observed in our screen is due to a single active component, but rather due to an additive or synergistic interaction between evodiamine, limonin, evocarpine, and likely other compounds. Such antimicrobial properties mediated by several phytobiotics are not uncommon in plants and likely extend to other species that were identified in our screen [96].

Our results reveal that *R. cordifolia* inhibited *M. luteus* only; however, previous studies have demonstrated its extensive antimicrobial properties against numerous clinically important bacterial pathogens, including gram-negative *P. aeruginosa* [62]. Its broad antimicrobial activity is possibly attributed to its ability to synthesize over 100 phytochemicals, many known for their extensive pharmacological activities [97]. Among *R. cordifolia*-producing phytochemicals, alizarin is one of at least 28 anthraquinones characterized by red color and potent antimicrobial properties [97,98]. Other notable *R. cordifolia* compounds with activity against *S. aureus* include rubiadin, xanthopurpurin, β-sitosterol glucoside, 1,2-dihydroxy-6-methoxyanthracene-9,10-dione, and pomolic acid [99]. Therefore, similar to *E. rutaecarpa*, *R. cordifolia* produces many phytobiotics that likely function together to enhance the collective antimicrobial activity of the plant extract.

The extract from *J. effusus*, commonly known as Soft Rush, exhibited a strong inhibitory effect against all gram-positive bacteria tested. Although the literature that exists on the antimicrobial effect of this TCM plant is scarce, our results are in agreement with previously published data revealing the strong antibacterial potency of its two extracts, dehydroeffusol and juncusol, against *S. aureus and B. subtilis* [50]. Additionally, a previous report revealed the inhibitory effect of *J. effusus*-derived phenanthrene compounds against *M. luteus* [51]. While only a few reports suggest the antimicrobial properties of *J. effusus*, several others noted its strong anti-inflammatory properties. For example, water extract from *J. effusus* exhibited anti-inflammatory properties explored as a potential application in periodontal diseases [100]. The anti-inflammatory activity of this herb was observed in several other studies [101,102]. Similarly to *J. effusus*, a few reports noted the antimicrobial properties of *D. nipponica*. In one study, *D. nipponica* extract, referred to by its Korean name, *Buchae-Ma*, slightly inhibited *B. subtilis*, *S. aureus*, *Proteus vulgaris*, and *Salmonella* Typhimurium in a disc diffusion assay [49]. However, in our study, we observed its efficacy only against *M. luteus*. Such differences in antimicrobial activities are not uncommon and are likely due to differences in extraction methods, which can result in variations in the concentration and composition of bioactive compounds.

One of the most studied TCM plants belongs to the *Artemisia* genus. Three of the extracts from two species, *annua* and *argyi*, identified in our screen belong to this genus. While our experiments revealed that the extracts from both species were effective only against *M. luteus*, others have observed their broad antimicrobial activity against a spectrum of gram-positive and -negative bacteria [103], fungi [104], viruses [105], and, of course, parasites [106]. Renowned for its exceptional antimalarial activity, an active component of *A. annua*, artemisinin is also known for its antimalarial, antibacterial, antiviral, antitumor, anti-inflammatory, and anti-fibrotic properties [107,108,109]. *A. argyi* extracts were also shown to be effective against an extended spectrum of gram-positive and -negative bacteria [45,110].

It may seem intuitive to leverage the antimicrobial activity of TCM plants to develop novel antimicrobials for human health; however, the potential applications to mitigate AMR extend beyond that of human applications. For example, the global efforts to ban the use of antibiotics as growth promoters in agriculture and reduce the use of antibiotics important in human medicine, necessitate the development of alternative strategies that would support sustainable food production and continue addressing the urgent and growing issue of AMR. Phytobiotics can be used in agriculture to maintain animal health and reduce the use of antibiotics. Studies on poultry have demonstrated the feasibility of such an approach with TCM plants [111,112]. For example, *A. annua* enhanced the health and growth of broilers [113]. Similar benefits were observed with *Agrimonia pilosa* [114]. *A. argyi* extracts were also shown to affect gut microbiota in mice, contributing to their growth [115], so the benefits of TCM plants could likely be extended to other animals. In fact, studies have also demonstrated the benefits of utilizing TCM plants in swine [116,117], beef [118], and fish [119]. Proven effective, TCM plants can be employed in food-producing animals; however, their application also comes with challenges that include cultural differences, a lack of standardization and quality control, potential toxicity, and, among other factors, supply issues [120].

### 3.3. Active Components of TCM Plant Extracts

Numerous studies have analyzed fractions from each of the 17 TCM plant species for active antimicrobial components, identifying over 100 compounds. Despite such a large number, it remains a surprise that, out of so many antimicrobial compounds, none have been successfully developed into drugs. The diverse chemical structures of the active components suggest they target a broad range of pathways and mechanisms, potentially distinct from those of conventional antibiotics. Almost all purified active components show favorable chemical properties according to Lipinski’s Rule of Five, indicating good potential for oral availability and intestinal absorption. However, given their potential toxicity at therapeutic concentrations and the possible synergistic effects between different active components suggest that their optimal use may remain within herbal medicine, particularly for gastrointestinal or mild infections.

Other notable and potential uses of the purified components include targeting of the resistance mechanisms, making them promising candidates for combinatorial use with conventional antibiotics. Several studies suggest the role of phytochemicals in targeting bacterial mechanisms mediating antibiotic resistance. For example, coumarin derivatives synergized with fluoroquinolone and aminoglycoside antibiotics against *S. aureus* and *E. coli* [121]. Several compounds structurally related to coumarin, including p-coumaric acid and quercetin, were previously extracted from *T. farfara* [67]; therefore, it is likely that the extract from this plan also synergizes with antibiotics.

While hierarchical clustering revealed ten different bins with a Tanimoto similarity score cutoff of 0.70, almost all the compounds within each bin come from the same plant species, with the exception of bins 7 and 8, which have compounds derived from two different *Artemisia* species, *annua* and *argyi*. Such species-specific structural conservation suggests their shared mechanisms of action and common targets, likely contributing to the overall antimicrobial activity of the extracts. Among the different bins, we found structural scaffolds that are known for their antimicrobial properties. For example, bin 1 contains prenylated flavonoid derivatives characterized by its chromane skeleton fused with an extended prenylated aromatic system. Interestingly, prenylated flavonoids are known for their antimicrobial properties [122]. A single plant species, such as *Glycyrrhiza uralensis*, can produce at least four structurally similar compounds, which likely enhances the antimicrobial potency of its extract. Similarly, bin 6 also contains compounds with similar structures derived from the same plant species: *E. rutaecarpa.*

Collectively, the extracts from the 17 plant species identified in our screen exhibit a broad range of antimicrobial activities against the three gram-positive organisms: *M. luteus*, *S. aureus*, and *S. epidermidis*. Many active components likely function synergistically, and others exhibit a strong antimicrobial potential when used alone or in combination with conventional antibiotics. Our study did not identify TCM plants for their novel antimicrobial indication, except for *E. latifolius*, as this was not our focus given centuries of their use for medicinal purposes, including infectious diseases. However, our results provide a robust basis for future investigation of the 17 TCM plant species for their potential human and animal health applications, particularly combating AMR bacteria and exploring synergistic therapies with existing antibiotics.

## 4. Materials and Methods

### 4.1. TCM Plant Extracts

The 664 crude plant extracts were provided by the Developmental Therapeutics Program at the NCI Natural Products Branch. Guangxi Botanical Garden of Medicinal Plants (GBGMP) in Nanning, China. The collection includes extracts from 132 authenticated plant species used in TCM. The library was provided in eight 96-well plates with 0.5 mg of plant extract per well. The extracts were reconstituted with 100 µL of DMSO (BP231-1, Fisher Bioreagents, Pittsburgh, PA, USA) added to each well, resulting in a 5 mg/mL stock solution. After reconstitution, all plates were stored at −80 °C.

### 4.2. Bacteria and Culture

*E. coli* DH5α (DC226), *M. luteus* SK58 (HM-114), *S. epidermidis* BCM0060 (HM-140), and *S. aureus* 12600 were inoculated fresh from −80 glycerol stock into 5 mL of sterile Luria–Bertani (LB) broth (Lennox, Apex Bioresearch Products, Genesee, El Cajon, CA, USA) and incubated at 37 °C with shaking at 220 RPM for 16 h.

### 4.3. Disk Diffusion Assay

Bacteria were swabbed uniformly across fresh tryptic soy agar (TSA, Difco, Sparks, MD, USA) plates using a sterile cotton swab and allowed to dry. Sterile 6 mm paper discs (Whatman, Buckinghamshire, UK) were arranged in a 3 × 4 grid on each plate, allowing for the testing of 12 extracts per plate. A 25 µL (25 µg total extract) volume of each extract diluted 1:4 from stock DMSO was aliquoted onto each disc. An appropriately diluted DMSO vehicle was used as a negative control. After the discs had dried, the plates were inverted and incubated overnight at 37 °C. Zones of inhibition (ZOIs) were measured and recorded in mm to assess the antimicrobial activity against the bacterial strains. The whole screen was repeated with *M. luteus*, and all hits were further confirmed using *M. luteus*, *E. coli*, *S. epidermidis*, and *S. aureus*.

### 4.4. Plate Dilution Assay

Serial dilutions were performed in a 96-well plate (GenClone, El Cajon, CA, USA) to test a select extract. A 20 μL volume of extract diluted to 1 μg/μL was added to 225 μL of LB. A 100 μL aliquot of the dilution was added to the first column of a 96-well plate. A series of 1:1 serial dilutions was performed by transferring 50 μL to the adjacent column. A 50 μL volume containing target bacteria was added to each well. Optical density at 600 nm (OD600) was measured every hour for 14 h using a Tecan Infinite M Nano^+^ (Männedorf, Switzerland) microplate reader to monitor growth.

### 4.5. Phylogenetic Analysis

The phylogenetic tree was generated based on a single gene: ribulose biphosphate carboxylase/oxygenase large subunit (RuBiSCO). Protein sequences were downloaded from the NCBI database and aligned using Mafft version 7 via the Bacterial and Viral Bioinformatics Resource Center’s (BV-BRC) website [123,124]. The .nwk tree output from BV-BRC was then visualized using the Interactive Tree of Life (iTol) version 5 [125].

### 4.6. Compound Structure Similarity

SMILES strings for each CID were retrieved from PubChem and uploaded to ChemMine tools [126]. Hierarchical clustering was performed based on Tanimoto Scores calculated for all compounds, and the results were visualized using a single-linkage method to generate a distance matrix heatmap. The resulting .tre output from ChemMine was further visualized using iTol (version 6) [127]. Binning was conducted with a similarity cutoff of 0.7. Compounds that clustered together are indicated by brackets. The corresponding structures obtained from ChemMine and CIDs are presented in Figure S4 and Table S3.

### 4.7. Lipinski Rule of Five

The partition coefficient (logP) was predicted using the ALOGPS 2.1 program at the Virtual Computational Chemistry Laboratory [128,129]. CIDs and SMILES strings were retrieved from PubChem. If no CID was available, the closest similar structure was used. Compounds that satisfied the Lipinski Rule of Five had MW < 500, 10 > HBA, 5 > HBD, and logP < 5.

### 4.8. Data Analysis

Growth curves were generated using GraphPad Prism 10.1.1 software (Boston, MA, USA).

## Figures and Tables

**Table 1:** Zones of inhibition in disc diffusion assay. Zones of inhibition (mm) produced by 25 extracts against representative gram-negative and gram-positive bacteria. The 25 extracts represent 17 unique plant species. A ZOI of 6 mm indicates no inhibition, as 6 mm corresponds to the diameter of the paper disc. * For *M. luteus*, the shown measurements represent the largest detected ZOI from among three replicates.

			Zone of Inhibition (mm)			
Spot #	Genus	Species	M. luteus *	*E. coli*	* S. aureus *	* S. epidermidis *
20	* Tussilago *	* farfara *	10	---	---	---
27	* Asarum *	* heterotropoides *	10	---	12.6	---
33	* Evodia *	* rutaecarpa *	17.6	---	11.6	10.8
42	* Rubia *	* cordifolia *	11.6	---	---	---
55	* Dioscorea *	* nipponica *	9.7	---	---	---
83	* Dioscorea *	* nipponica *	11.6	---	---	---
95	* Echinops *	* latifolius *	10.9	---	11.8	---
201	* Artemisia *	* annua *	19.4	---	---	---
245	* Glycyrrhiza *	* uralensis *	12.6	---	11.7	11.6
248	* Areca *	* catechu *	10.6	---	---	---
251	* Artemisia *	* argyi *	11	---	---	---
275	* Juncus *	* effusus *	10.2	---	11.2	---
285	* Artemisia *	* argyi *	8.4	---	---	---
395	* Juncus *	* effusus *	13	---	13.2	11.8
396	* Lithospermum *	* erythrorhizon *	14	9.5	13.9	14.8
413	* Sophora *	* flavescens *	12.2	---	11.2	11.4
415	* Juncus *	* effusus *	11.2	---	11.4	---
416	* Rubia *	* cordifolia *	12	---	---	---
424	* Evodia *	* rutaecarpa *	19	---	9.8	---
456	* Agrimonia *	* pilosa *	11.4	---	12.2	10.6
460	* Lithospermum *	* erythrorhizon *	12.6	---	10.2	12.4
694	* Salvia *	* miltiorrhiza *	12.2	---	9.81	15.6
704	* Siegesbeckia *	* orientalis *	9.4	---	---	---
727	* Evodia *	* rutaecarpa *	18.4	---	11	11.8
749	* Pogostemon *	* cablin *	21.4	---	---	---

## Data Availability

All data generated or analyzed during this study are included in this published article and its Supplementary Materials. Any additional information will be provided upon request.

## Supplementary Materials

The following supporting information can be downloaded at: https://www.mdpi.com/article/10.3390/antibiotics13121220/s1, Figure S1: Results of the screen. Images of all hits against *M. luteus* and *E. coli*. Figure S2: Confirmation screen. Images of all 25 original hits tested against *M. luteus*, *S. epidemidis*, *S. aureus*, and *E. coli*. Figure S3: Compound structure similarity tree. Brackets indicate compounds that cluster together into ten bins with a Tanimoto score similarity cutoff of 0.7. Corresponding CIDs are presented in Table S2. Figure S4. Chemical structures of compounds from each of the ten bins. Corresponding CIDs are presented in Table S2. Structures were obtained from ChemMine. Table S1: Twenty-five hits representing 17 unique TCM plants. The initial screen was repeated using *M. luteus* (Original Screen) and confirmed with individual hits, using *E. coli*, *S. aureus*, *M. luteus*, and *S. epidermidis*. Table S2: Isolated antimicrobial compounds and their activity against bacteria. The antimicrobial effect of each compound is highlighted in gray and marked with a “+”. Corresponding compound ID (CID) numbers for each compound (or its derivative) were obtained from PubChem. NA—Not available. The table does not represent a comprehensive analysis of all studies. Table S3: Lipinski Rule of Five. Simplified Molecular Input Line Entry System (SMILES) strings for each antimicrobial compound were retrieved from PubChem. Molecular weight (MW), hydrogen bond acceptor (HBA), and hydrogen bond donor (HBD) were predicted using ChemMine based on the CID-unique SMILES. The partition coefficient (logP) values were predicted using the ALOGPS 2.1 Program. Compounds without available CIDs were either marked as not available (NA), or the most structurally similar compound was used, denoted by an asterisk (*). Highlighted cells represent metrics that violated Lipinski’s Rule of Five. Of the 78 unique compounds analyzed, 14 did not meet the rule’s criteria. Structures for the corresponding CIDs were acquired from PubChem.
