# Supplementary figures and images for: A Screen of Traditional Chinese Medicinal Plant Extracts Reveals 17 Species with Antimicrobial Properties
Source: Antibiotics (Basel). 2024 Dec 17;13(12):1220. doi: 10.3390/antibiotics13121220 (PMC11726858; doi:10.3390/antibiotics13121220)

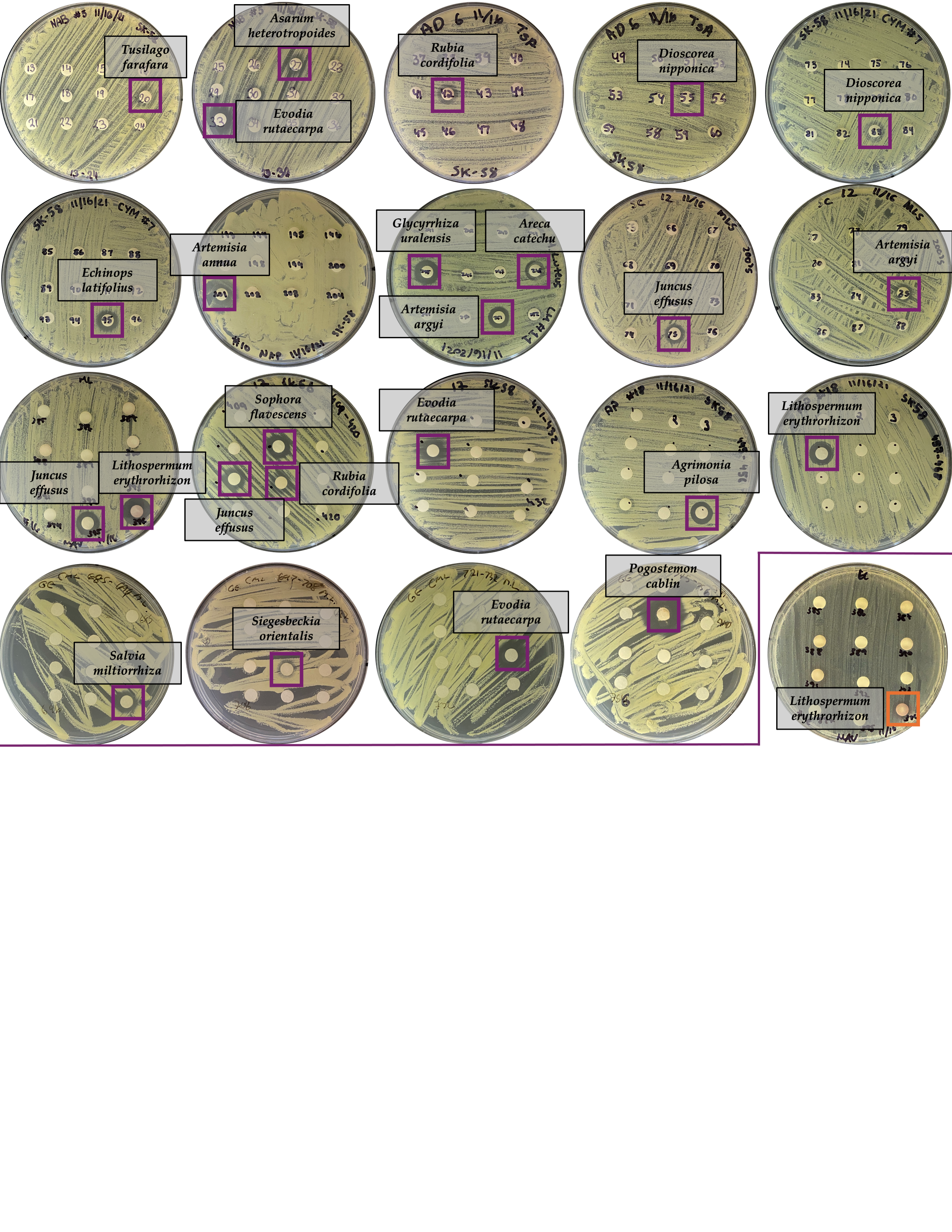

Supplement: Supplementary file 1 [file antibiotics-13-01220-s001.zip › Figure_S1.tiff]

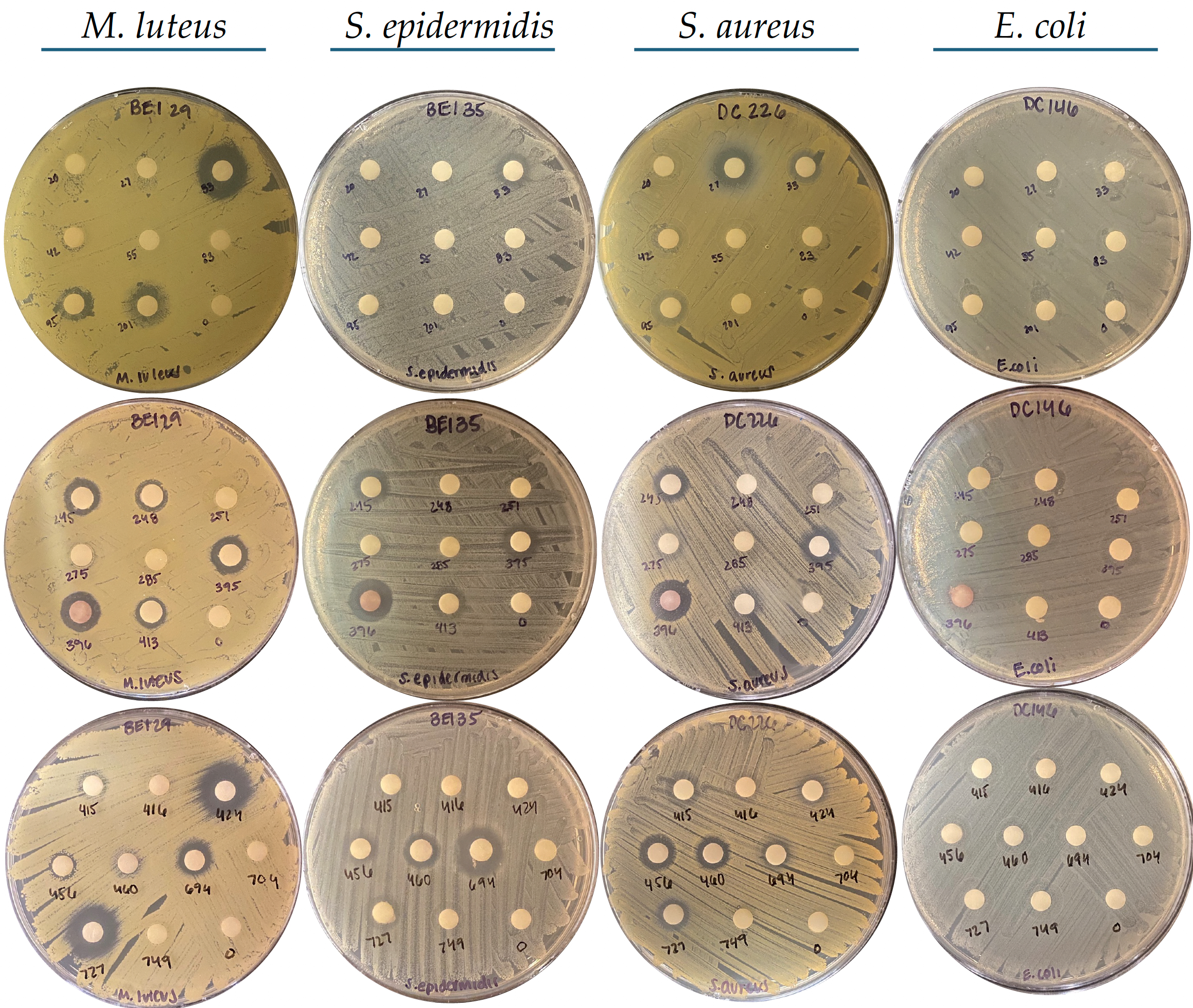

Supplement: Supplementary file 1 [file antibiotics-13-01220-s001.zip › Figure_S2.jpg]

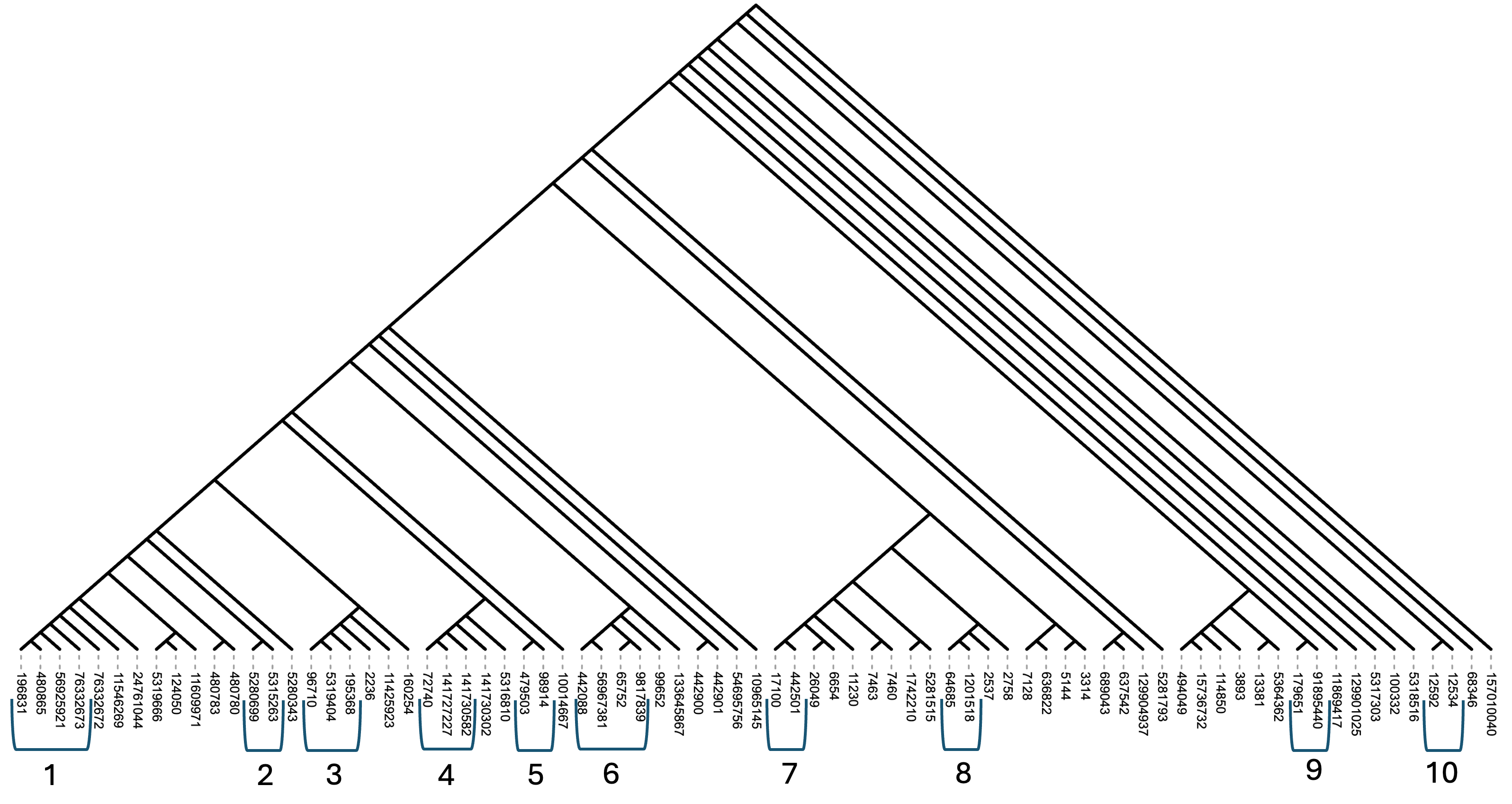

Supplement: Supplementary file 1 [file antibiotics-13-01220-s001.zip › Figure_S3.jpg]

# Bin 1

196831

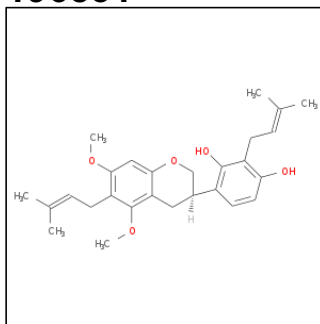

480865

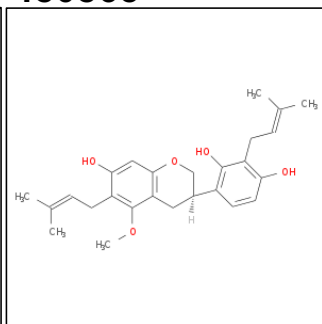

56925921

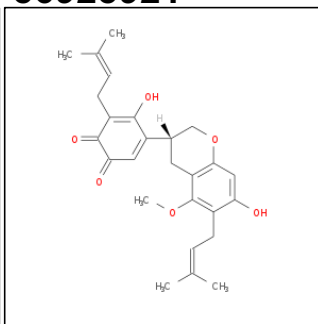

76332673

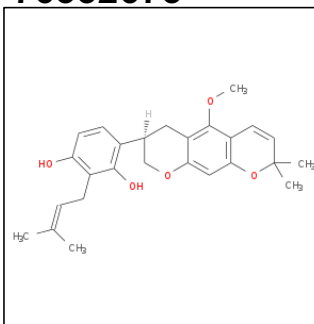

# Bin 2

**5280699**

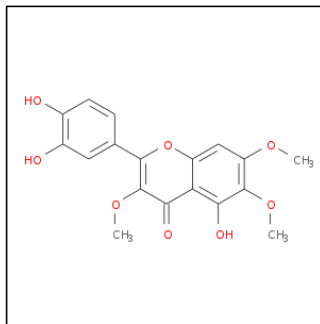

**5315263**

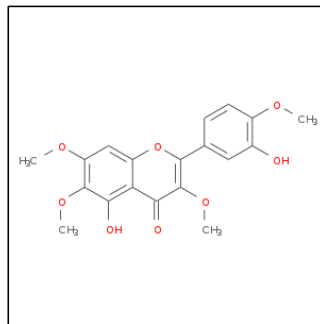

# Bin 3

96710

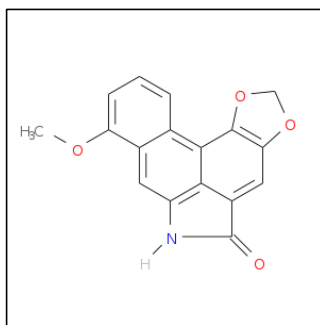

5319404

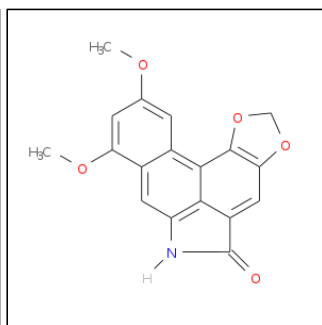

195368

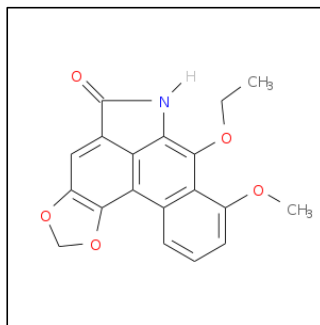

# Bin 4

**72740**

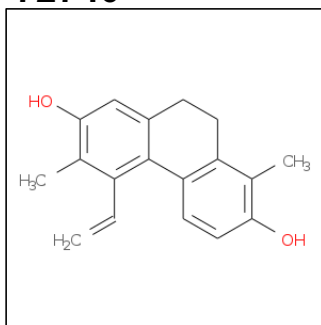

**141727227**

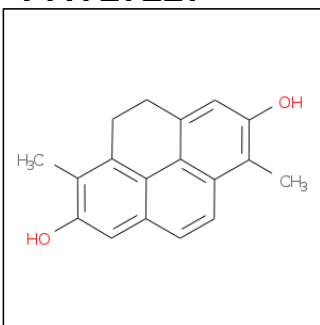

**141730582**

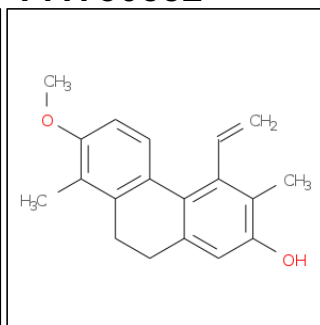

# Bin 5

**479503**

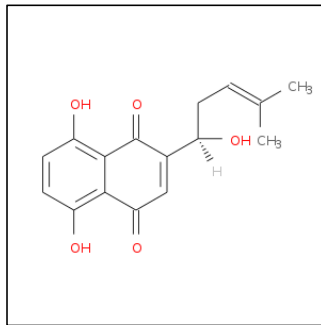

**98914**

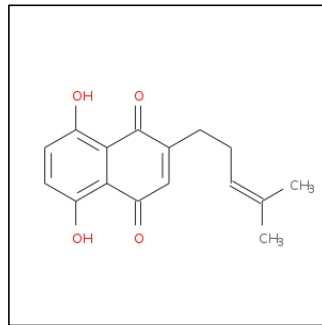

# Bin 6

442088

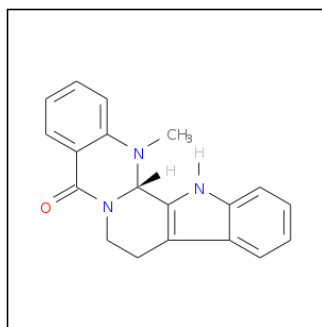

56967381

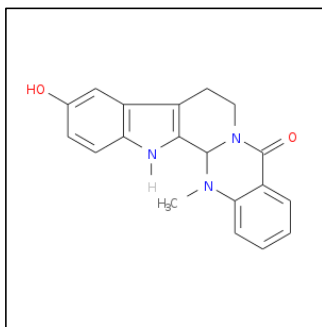

65752

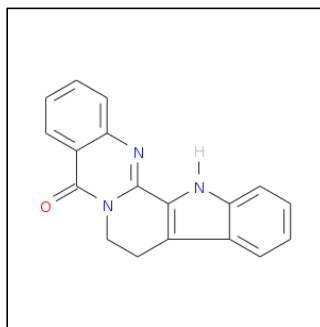

9817839

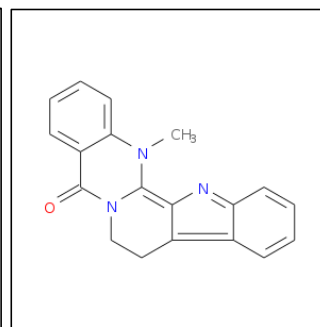

# Bin 7

17100

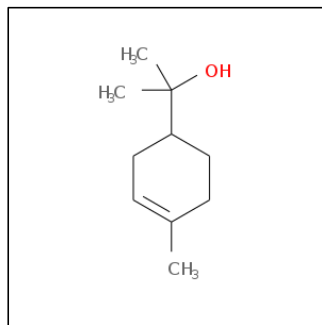

442501

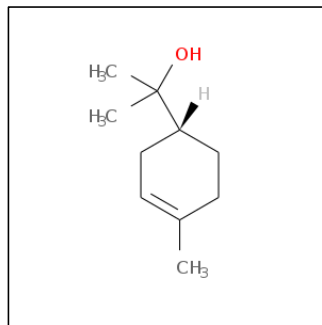

# Bin 8

64685

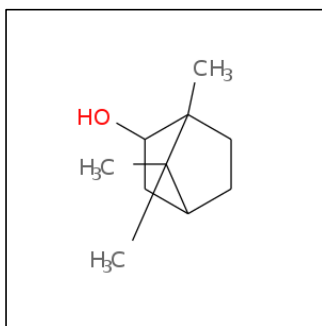

1201518

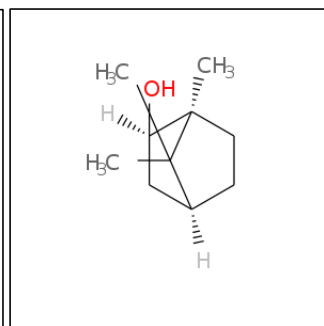

# Bin 9

**179651**

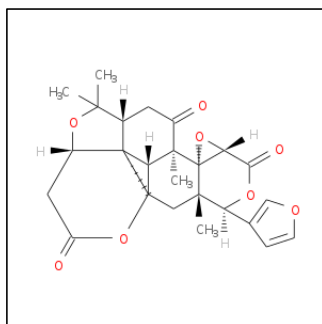

**91895440**

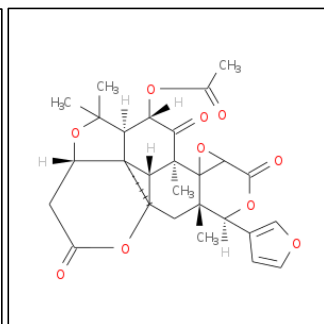

# Bin 10

**12592**

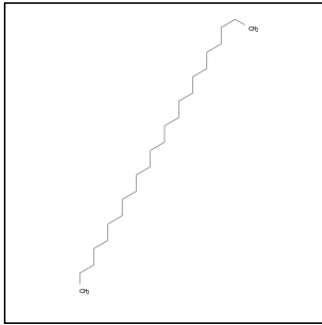

**12534**

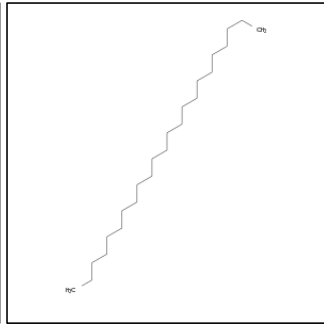

Supplement: Supplementary file 1 [file antibiotics-13-01220-s001.zip › Figure_S4_new.pdf]
